# Cross-Cultural Adaptation of the Breast Cancer and Lymphedema Symptom Experience Index in Bengali

**DOI:** 10.1177/10436596251345338

**Published:** 2025-06-28

**Authors:** Vincent Singh Paramanandam, Elizabeth Dylke, Mei R. Fu, Anuradha Daptardar, Manali Kamat, Sarika Mahajan, Sharon Kilbreath

**Affiliations:** 1 Macquarie University, Sydney, New South Wales, Australia; 2 The University of Sydney, New South Wales, Australia; 3 University of Missouri-Kansas City, USA; 4 Tata Memorial Hospital, Mumbai, India

**Keywords:** breast cancer, lymphedema, Breast Cancer–related Lymphedema and Symptom Experience Index (BCLE-SEI), symptoms, patient-reported outcome measures (PROM)

## Abstract

**Introduction:**

Breast cancer–related lymphedema is associated with a myriad of distressing symptoms and significantly impacts survivors’ quality of life. The Breast Cancer–related Lymphedema Symptom Experience Index (BCLE-SEI) is a validated patient-reported outcome measure (PROM), but is unavailable in Indian languages. This study aimed to cross-culturally adapt the BCLE-SEI into the Bengali language.

**Method:**

Following established guidelines, the BCLE-SEI was translated, back-translated, and validated in 168 Bengali-speaking women with or at risk of lymphedema.

**Results:**

Content validity was confirmed by ≥98% of participants. Exploratory factor analysis identified two factors in symptom occurrence and distress subscales, explaining 43.9% and 51% of the variance, respectively. Internal consistency (α) was >0.85, and construct validity was supported with over 50% of predefined hypotheses met. Test–retest reliability (intraclass correlation coefficient [ICC_2,1_]) was .46 (95% confidence interval [CI] = [–0.2, 0.8], *p* = .076).

**Discussion:**

The BCLE-SEI-Bengali is a reliable and valid PROM for assessing lymphedema-related symptoms for Bengali-speaking women.

## Introduction

Breast cancer is the leading cancer in women globally and in India (Bray et al., 2024). Breast cancer–related lymphedema (BCRL) remains the most debilitating and long-term adverse effect of breast cancer treatment (DiSipio et al., 2013; Fu et al., 2015). Treatment-related risk factors of BCRL include late cancer stage, axillary lymph node dissection (ALND), number of lymph nodes removed, mastectomy, radiotherapy, and chemotherapy (Lin et al., 2021; Wu et al., 2019). Modifiable lifestyle risk factors of BCRL are identified to include high body mass index (BMI) and skin trauma (Fu et al., 2024; Lin et al., 2021; Wu et al., 2019). More than 20% of patients who have undergone ALND have developed BCRL (DiSipio et al., 2013). Although the rate of ALND has been reduced significantly in developed nations due to changes in treatment protocol (Fancellu et al., 2025; Luzaic et al., 2025), it is not completely avoidable, and a considerable percentage of women in India undergo ALND as a part of their breast cancer management due to delayed diagnosis of breast cancer (Anand et al., 2022; Bhargavan et al., 2024; Wadasadawala et al., 2024). BCRL is associated with a myriad of signs and symptoms, including swelling, heaviness, tightness, pain, and discomfort, which can lead to distress and a poor quality of life (QOL; Huang et al., 2022; Macdonald et al., 2024; Togawa et al., 2021). Assessing and monitoring BCRL-related symptom experience is important to providing patient-centered care (Fu et al., 2015; Gursen et al., 2021; Hayes et al., 2022).

The management of BCRL presents a unique challenge due to the subjective nature of its associated symptoms. The variability in individual experiences necessitates the use of patient-reported outcome measures (PROMs) to accurately capture and assess these symptoms (Fu et al., 2015; Qiu et al., 2024). Patients’ symptom experiences not only influence their QOL but also guide clinical decision-making, treatment planning, and the overall approach to patient-centered care (Fu et al., 2015; Gursen et al., 2021; Sackey et al., 2015; Togawa et al., 2021). Consequently, assessing and monitoring BCRL-related symptom experiences are pivotal in tailoring interventions that align with the specific needs and preferences of patients. This focus on individualized and patient-centered care is supported by various studies that emphasize the importance of integrating patient perspectives into clinical practice (Fu et al., 2015; Gursen et al., 2021; Hayes et al., 2022; Svensson et al., 2020). Assessing BCRL-related symptoms can help health care providers to make treatment decisions, enhance the effectiveness of treatment strategies, improve patient satisfaction, and ultimately foster a comprehensive approach to cancer survivorship (Fu et al., 2018, 2021).

PROMs are effective tools for measuring BCRL-related symptoms. There are two PROMs developed specifically to measure and monitor the symptoms related to BCRL: The Breast Cancer and Lymphedema Symptom Experience Index (BCLE-SEI) and Lymphedema Symptom Intensity and Distress Survey-Arm (LSIDS-A; Cachero-Rodriguez et al., 2022; Fu et al., 2015; Li et al., 2016; Ridner & Dietrich, 2015; Shi et al., 2016). However, they are not available in Indian languages (Paramanandam et al., 2021). Validated PROMs available in Indian languages that include questions regarding arm symptoms in Bengali, the third common language, are embedded within broader QOL questionnaires such as European Research and Treatment Committee Quality of Life Breast Cancer modules (EORTC-QLQ-C30 and BR23) and do not provide details about the severity of a range of symptoms described in association with lymphedema (Oliveira et al., 2015; Parmar et al., 2005). A validated PROM would, therefore, be beneficial in assessing and monitoring the symptom experience of BCRL in Indian languages.

The BCLE-SEI captures the presence of BCRL-related symptoms and symptom distress. In comparison to LSIDS-A and EORTC PROMs, the advantages of the BCLE-SEI include its role in detecting BCRL cases using symptom counts (Fu et al., 2015), machine learning prediction (Fu et al., 2018), and monitoring treatment effectiveness (Fu et al., 2021). The BCLE-SEI was initially developed in English (Fu et al., 2015) and was later cross-culturally adapted (translated and validated) into Chinese (Shi et al., 2016) and Spanish (Cachero-Rodriguez et al., 2022). Psychometric properties of the BCLE-SEI have been investigated in English, Chinese, and Spanish versions, particularly for structural validity, internal consistency, test–retest reliability, and construct validity (Cachero-Rodriguez et al., 2022; Fu et al., 2016; Paramanandam et al., 2021; Shi et al., 2016). The aim of this study was to cross-culturally adapt the BCLE-SEI into the Bengali language, one of the most commonly spoken languages in India, and evaluate its psychometric properties.

## Method

### Ethical Considerations

Ethical clearance was obtained from the institutional ethics board of Tata Memorial Hospital, Mumbai, India (IEC/1017/1946/002). The study was registered prospectively in the clinical trial registry of India (CTRI/2017/11/010326). Written informed consents were obtained from all the participants.

### Design and Method

Two-stage cross-cultural adaptation of the BCLE-SEI questionnaire was undertaken following a standardized process (Eremenco et al., 2018; International Test Commission, 2017). Permission from the author of the English version BCLE-SEI was obtained before the initiation of the study.

### Stage 1: Translation and Equivalence

The English version of the BCLE-SEI was translated into the Bengali language by two independent bilingual translators who were health care professionals and knowledgeable in the cultural differences between the source population, English (US), and the target population, Indian Bengali, and with an adequate understanding of the construct of the symptom experience. These translated copies were then synthesized into a single consolidated questionnaire by a bilingual coordinator in collaboration with the translators and through a consensus process (Eremenco et al., 2018).

This consolidated Bengali version (i.e., the BCLE-SEI-Bengali) was then back-translated independently by two certified bilingual translators to English, who had not seen the original English version. These back-translated English versions were synthesized by a bilingual coordinator and the translators through a consensus process (Eremenco et al., 2018). This consolidated version was evaluated by an expert committee comprising lymphedema therapists, physiotherapists, and psychologists working in cancer care for more than 5 years for conceptual equivalence to the English version. In addition, eight women with BCRL reviewed the consolidated version, that is, the BCLE-SEI-Bengali, for its face validity.

### Stage 2: The Validation Study

#### Participants

A total of 170 women, who completed breast cancer treatment and were with or without a BCRL diagnosis, participated in this study. The participants were ≥18 years of age, had undergone treatment for unilateral breast cancer, were not reporting significant cognitive impairment or lymphedema due to reasons other than breast cancer treatment, and were able to complete the self-reported BCLE-SEI-Bengali without assistance. After women signed the informed consent to participate, the participants’ characteristics, such as date of birth, hand dominance, menopausal status, and past lymphedema-related information, were collected through structured interviews. Other clinical and breast cancer treatment-related information was obtained from the electronic medical records and case files.

#### Physical Measurement

Physical measures, including height and weight, and measures indicative of lymphedema using bioimpedance and arm circumferences were undertaken. The participants’ heights were measured using a wall-mounted stadiometer (Libra, India) to the nearest 0.5 cm, and their weights were measured using a digital weighing scale (Sknol, model number 7281) to the nearest 0.1 kg. The bioimpedance measurements were performed with an SFB7 bioimpedance spectroscopy device (Impedimed Ltd., Brisbane) using the equipotential method with an established protocol (Cornish et al., 1999). The interarm bioimpedance ratio (BIS ratio) was calculated, and two thresholds were used to determine BCRL based on the normative mean and two and three standard deviations (≥mean + 2*SD* and ≥mean + 3*SD*). Arm circumference measurements were taken while participants sat upright with their arms elevated and supported at 90^0^ shoulder flexion. Circumference measurements were taken at the ulnar styloid and 10 cm intervals proximally to 40 cm using an inelastic tape measure and recorded to the nearest 1.0 mm. Each arm’s volume was calculated using the truncated cone formula from the arm circumference measurements (Brorson & Hoijer, 2012). Relative (percentage) arm volume differences (RAVD) between the at-risk and unaffected arms were then calculated (Bundred et al., 2020), and cases with the RAVD between the unaffected and the at-risk or the BCRL arms (≥5% and ≥ 10%) were identified.

#### Patient-Reported Outcome Measures

Participants completed the BCLE-SEI-Bengali and the EORTC QLQ-C30 and B23 in Bengali without assistance. The time taken to complete the BCLE-SEI-Bengali was recorded. In addition, patients were interviewed with six questions regarding the contents of the BCLE-SEI-Bengali to evaluate its relevance, comprehensibility (clarity), and comprehensiveness (all relevant items are present) to their experience with BCRL (Supplementary File).

#### BCLE-SEI-Bengali

The BCLE-SEI-Bengali has 34 items divided into two subscales: Subscale 1: symptom occurrence, which includes severity, and Subscale 2: symptom distress (Fu et al., 2015, 2021, 2024). The symptom occurrence subscale measures 24 symptoms, assessed through 27 items, related to BCRL and its severity. The first four items are considered as one. To score, the presence or absence of the symptoms can either be evaluated as categorical data with a “Yes or No” response at the individual item level or as a continuous score by counting all the “Yes” responses. The severity of the symptoms is measured using a 5-point Likert-type scale, and the overall score ranges from 0 to 96 (Fu et al., 2015, 2021), with higher scores reflecting more severe symptom experiences. The symptom distress subscale measures the negative impact of symptoms related to the BCRL. Symptom distress is measured using a 5-point Likert-type scale, and the overall score ranges from 0 to 128, with a higher score reflecting more severe symptom-related distress. There are six proposed dimensions in the symptom distress subscale: physical functional or daily living function, social, sleep disturbance, sexuality, emotional, and attributive dimensions (Cachero-Rodriguez et al., 2022; Fu et al., 2015, 2021; Shi et al., 2016).

The psychometric properties of the English, Chinese, and Spanish versions of the BCLE-SEI questionnaires have been established (Cachero-Rodriguez et al., 2022; Fu et al., 2015, 2016, 2021; Shi et al., 2016). Exploratory factor analysis (EFA) of the Chinese version of BCLE-SEI revealed five dimensions in each of the two subscales, whereas the Spanish version revealed a unidimensional dimension for the symptom occurrence subscale and two dimensions for the symptom distress subscale. Cronbach’s alpha coefficient of internal consistency for the questionnaire and the dimensions ranged from .92 to .98 in both versions. Spearman’s correlation coefficient (Rho) for the test–retest reliability of the BCLE-SEI Chinese ranged from .66 to .71, whereas the correlation of the BCLE-SEI Spanish ranged from .78 to .87. Construct validity of the BCLE-SEI Chinese questionnaire was established through correlations within the dimensions of BCLE-SEI Chinese and dimensions of the Short-Form Health Survey (SF-36) (*r* = .35–.93). The BCLE-SEI Chinese and Spanish questionnaires were therefore found to have sufficient construct validity.

#### EORTC QLQ-C30 B23

The EORTC QLQ-C30 BR23 is a commonly used and most frequently cross-culturally adapted breast cancer-specific QOL questionnaire (Ho et al., 2018; Nguyen et al., 2015). EORTC QLQ-C30 BR23 is a modular questionnaire comprising a core module, QLQ-C30, and a supplementary module, BR-23. The core module consists of 30 QOL items related to the wider range of cancer populations, and the supplementary module consists of 23 QOL items specific to the breast cancer population. The core module includes physical, role, cognitive, emotional, and social multi-item functional scales; fatigue, pain, and emesis multi-item symptom scales; six single symptom items, a financial burden and a global QOL item (Aaronson et al., 1993; Parmar et al., 2005). The supplementary breast-specific module includes body image, future perspective, and sexuality multi-item scales and four symptom scales, including arm symptoms, breast symptoms, hair loss, and the side effects of the treatment (Parmar et al., 2005; Sprangers et al., 1996).

The EORTC QLQ-C30 BR23 has good psychometric properties (Aaronson et al., 1993; Maratia et al., 2016; Sprangers et al., 1996). This questionnaire has been cross-culturally adapted and validated in Indian languages, including Bengali (Parmar et al., 2005). Cronbach’s alpha for the internal consistency ranged from .61 to .96; the item scale correlation ranged from .63 to .93. The known group validity and responsiveness of this questionnaire have also been documented (Parmar et al., 2005).

A subgroup of 10 participants who were willing to attend 1 week after the initial testing underwent repeat measurements and filled out the questionnaire again to assess the test–retest reliability.

### Data Analysis

A sample size of 170 was considered adequate for the validation study based on five participants per item (5*34) in the BCLE-SEI-Bengali questionnaire (Anthoine et al., 2014). However, two of the participants had more than 50% of missing data; hence, they were excluded from data analysis. Descriptive statistics were used to analyze the demographic, clinical, and treatment-related variables. To analyze the content validity, the proportion of participants agreeing to relevance (relevant to the symptoms of BCRL), comprehensiveness (including all the relevant items), and comprehensibility (clear and understandable) was calculated. In addition, at least two authors reviewed the qualitative responses to the items. The threshold for content validity was set to 85% of the participants must agree that the items are relevant, comprehensive, and comprehensible.

The structural validity of the questionnaire was evaluated using the EFA. The adequacy of the sample size for the EFA was evaluated using the Kaiser–Meyer–Olkin (KMO) test, and >0.5 was considered acceptable. Barlett’s test of sphericity was used to determine whether the correlation was an identity matrix. The Pearson correlation matrix was calculated, and a method was used to extract factors from the 27-item symptom occurrence and 32-item symptom distress subscales of the BCLE-SEI questionnaire. The Hot-Deck Multiple Imputation in EFA was used to impute the missing values (Lorenzo-Seva & Van Ginkel, 2016). The suitability of the robust unweighted least squares method (RULS) was assessed prior to the analysis (Lorenzo-Seva & Ferrando, 2006). A Parallel Analysis (PA) method was used to estimate the number of factors to be extracted (Timmerman & Lorenzo-Seva, 2011). A Robust Promin oblique rotation was employed to aid interpretability. Items loading lower than 0.30 were dropped from the rotation matrix (Lorenzo-Seva & Ferrando, 2006).

The Cronbach’s alpha test was used to evaluate the internal consistency of the total scale, subscales, and dimensions. The intraclass correlation coefficient (ICC_2,1_) was calculated for the total scale and subscales scores obtained twice in 1-week intervals to evaluate the test–retest validity of the questionnaire. To evaluate the known-group discriminant validity, an independent Student’s *t* test was used to compare the total and the subscales scores between women who had BCRL and those who were at risk of BCRL. The construct validity of the questionnaire was evaluated through the generic hypothesis proposed a priori (hypothesis testing). To test the hypothesis, correlations between the dimensions’ scores of the BCLE-SEI questionnaire and the various scales’ scores of the EORTC-QLQ-C30 and BR23 questionnaire were examined using Spearman’s correlation coefficient. Subsequently, this correlation matrix was used to test the following hypotheses developed based on the Consensus-based Standards for Measurement Instruments (COSMIN) guidelines: (a) all the dimensions scores of the BCLE-SEI-Bengali are negative—higher scores are either severe symptoms or higher distress; (b) EORTC QLQ-C30 BR23 functional scales are positive, which means higher scores mean better function whereas the symptoms scales are negative that means higher scores means worst symptoms; (c) all the dimensions of the BCLE-SEI are, therefore, expected to have a negative correlation with EORTC QLQ-C30 BR23-functional scales and a positive correlation with the EORTC QLQ-C30 BR23-symptom scales; (d) correlations with instruments measuring similar constructs should be >.50; (e) correlations with instruments measuring related, but dissimilar constructs should be lower, that is, .30–.50; and (f) correlations with instruments measuring unrelated constructs should be <.30. It was determined that the construct validity of the questionnaire would have been met if 50% or more correlations met the abovementioned criteria.

The receiver operating characteristic (ROC) curve analysis was performed to evaluate the ability of the BCLE-SEI-Bengali to screen for the presence of BCRL. The ROC analysis was used to determine the area under the curve (AUC) and to determine the number of symptoms criterion value based on four thresholds commonly reported in the literature to determine BCRL: (a) BIS ratio ≥ mean + 2*SD*, (b) BIS ratio ≥ mean + 3*SD*, (c) RAVD ≥5%, and (d) RAVD ≥ 10%. The number of symptoms was calculated by counting the “Yes” responses to the items in the symptom occurrence subscale. The symptoms count criterion (cut-off value) for BCRL was determined using the Youden Index method and reported the sensitivity and specificity for the number of symptoms count criterion. The factor analysis was performed using the Factor program (Release 12.04.05 64-bits). The AUC curve analysis was performed using the MedCalc (Version 22.230 – 64-bits). All other analyses were performed using SPSS (Version 29.0.0.0 (241)).

## Results

### Participants

A total of 168 women with or at risk of BCRL were included in the final analysis. Table 1 presents the participants’ characteristics. Briefly, the participants’ mean age was 47.7 (±9.8). Forty-seven of the participants presented with BCRL based on clinical examination, and an additional eight participants reported having BCRL in the past. Women with BCRL had higher pathological grading of their cancer, reported a higher number of axillary node removals, and a higher proportion received regional lymph node radiation (supraclavicular node) than women without lymphedema. As expected, the baseline interarm impedance ratio and arm volume difference of the women with BCRL were higher than those without lymphedema.

**Table 1.** Participant and Breast Cancer Treatment Characteristics.

Characteristics	All (*n* = 168)	Without lymphedema (*n* = 121)	With lymphedema (*n* = 47)	*p* value
Age, years, *M* (*SD*)	47.7 (9.8)	47.9 (9.9)	47.2 (9.7)	.95^†^
Hand dominance—right, *n* (%)	163 (97)	117 (96.7)	46 (97.9)	.72*
BMI, kg/m^2^, *M* (*SD*)	27.58 (4.7)	26.5 (4)	27.5 (3.5)	.12^†^
WHR, *M* (*SD*)	0.91 (0.1)	0.91 (0.1)	0.92 (0.1)	.17^†^
*Affected side*				
Right, *n* (%)	76 (45.2)	56 (46.3)	20 (42.6)	.66*
*Pathology Grading*				
2, *n* (%)	31 (18.5)	24 (20.7)	7 (14.9)	.39*
3, *n* (%)	132 (78.6)	92 (79.3)	40 (85.1)	.39*
*Hormone receptor status*				
Estrogen positive, *n* (%)	119 (70.8)	88 (77.2)	31 (66)	.14*
Progesterone positive, *n* (%)	101 (60.1)	75 (65.2)	26 (55.3)	.24*
HER2 positive, *n* (%)	83 (49.4)	54 (47.4)	29 (61.7)	.72*
*Neoadjuvant chemotherapy*				
NACT, *n* (%)	84 (50)	56 (46.3)	28 (59.6)	.12*
*Arm measurements*				
Inter-arm impedance ratio, ohm, *M* (*SD*)	1.1 (0.2)	1 (0.2)	1.3 (0.3)	**<.001** ^†^
Relative arm volume difference, %, *M* (*SD*)	4.7 (12.3)	0.2 (6.9)	16.2 (15.4)	**<.001** ^†^
*Breast surgical procedures*				
Local Biopsy, *n* (%)	133 (79.2)	93 (76.9)	40 (85.1)	0.24*
Excision Biopsy, *n* (%)	10 (6)	10 (8.3)	0 (0)	0.63*
BCS, *n* (%)	54 (32.1)	37 (30.6)	17 (36.2)	0.49*
Mastectomy, *n* (%)	88 (52.4)	62 (51.2)	26 (55.3)	0.64*
Reconstruction, *n* (%)	4 (2.4)	3 (2.5)	1 (2.1)	0.89*
*Axillary surgery* (*n* = 168)				
Number of nodes removed, *n* (%)	16.7 (7.7)	15.33 (7.5)	20.26 (7.3)	**<.001** ^†^
Number of nodes involved, *n* (%)	2.26 (5)	1.76 (3.6)	3.53 (7.3)	.12^†^
*Adjuvant Chemotherapy*				
Chemotherapy, *n* (%)	143 (85.1)	100 (82.6)	43 (91.5)	.15*
*Hormone therapy, n* (%)	118 (70.2)	87 (71.9)	31 (66)	.45*
*Radiotherapy*	147 (87.5*)*	103(85.1)	44 (93.6)	.14*
Breast, *n* (%)	100 (59.5)	75 (62)	25 (53.2)	.30*
Chest wall, *n* (%)	50 (29.8)	29 (24.0)	21 (44.7)	**.01** *
Axilla, *n* (%)	1 (0.6)	1 (0.8)	0 (0)	.53*
Supraclavicular, *n* (%)	117 (69.6)	81 (66.9)	36 (76.6)	.22*

*Note.* BMI = body mass index; WHR = waist to hip ratio; NACT = neoadjuvant chemotherapy; BCS = breast conservation surgery; n = number; SD = standard deviation; † = independent *t* test; * = X^2^ test; bold = statistically different.

### Content Validity

Sufficient content validity of BCLE-SEI-Bengali was established. A high proportion of participants, 98% with or at risk of BCRL, agreed that the items and the scoring system were relevant to BCRL-related symptom experience. Likewise, 97% of the participants with BCRL and 99% at risk of BCRL said that the BCLE-SEI-Bengali’s items and instructions were easy to understand (comprehensible). Three participants mentioned that a few of the items were challenging, for example, “soreness,” but understandable. In addition, comprehensiveness (completeness) of the BCLE-SEI Bengali was agreed upon by 94% of the participants with BCRL and 98% at risk of BCRL. One percent of participants suggested various possible additions, such as “difficulty sleeping on the side with Lymphedema,” when asked about additional symptoms or experiences they had with BCRL. Two participants wanted the sexual distress due to BCRL to be deleted when the participants were asked whether they wanted to modify or delete any of the items in the BCLE-SEI-Bengali questionnaire. After in-depth consideration of the responses, the authors decided that no items needed to be added or deleted due to the infrequent nature of the suggested changes.

### Structural Validity

Inspection of the Pearson correlation matrices showed that all variables had at least one correlation coefficient ≥.30 in both subscales. The overall KMO measure was 0.8 for the symptom occurrence and 0.86 for the distress subscale, with individual KMO measures all greater than 0.7, indicating sampling adequacy. Bartlett’s test of sphericity was statistically significant (*p* < .0005), indicating that the data was likely factorizable.

The RULS revealed that seven factors in each subscale had eigenvalues greater than 1, which explained 68% and 67.9% total variances for the occurrence and distress subscales, respectively. However, the parallel analysis based on minimum rank factor analysis (Timmerman & Lorenzo-Seva, 2011) proposed two factors for the occurrence and three factors for the distress subscales, respectively. A Robust Promin oblique rotation showed that the two and three factors for the occurrence and distress subscales, respectively, are conceptually meaningful. However, the rotated solution did not produce a “simple structure” for occurrence and distress subscales.

The rotated loading matrix showed that Item 15 of the occurrence subscale, “Blistering in the affected limb,” did not load well on both factors (<0.253). Item 22, “pocket of fluid developed,” also had low loading (0.310), with both these items semantically similar in Bengali and showed high skewness and kurtosis, low normed Measure of Sampling Adequacy (MSA) and low communality. Item 15 of the occurrence subscale, therefore, was dropped, and the EFA was rerun, which produced a simple structure with two factors: (a) tissue changes and (b) Movement restriction (Table 2). The two-factor solution for the occurrence subscale explained 43.9% of the total variance.

**Table 2.** Factor Loadings for BCLE-SEI-Bengali.

Symptom occurrence subscale			Symptom distress subscale		
Occurrence Items	Tissue changes	Movement restrictions	Distress Items	Physical-functional distress	Psychosocial distress
Pain	0.086	**0.585**	Cooking	**0.823**	0.058
Soreness	0.128	**0.607**	Cutting food with a knife	**0.813**	-0.051
Aching	0.082	**0.722**	Writing/typing	**0.653**	-0.041
Tenderness	-0.008	**0.581**	Cleaning the house	**0.848**	0.004
Arm or hand swelling	0.086	**0.439**	Vacuuming	**0.819**	0.043
Breast swelling	-0.077	**0.481**	Doing the laundry	**0.534**	0.161
Chest wall swelling	-0.164	**0.43**	Bathing or showering	**0.563**	-0.055
Firmness in the affected limb	-0.084	**0.693**	Taking care of children	**0.555**	0.026
Tightness in the affected limb	-0.021	**0.622**	Gardening	**0.449**	0.098
Heaviness in the affected limb	-0.042	**0.697**	Getting dressed	**0.68**	-0.175
Toughness or thickness of skin in the affected limb	0.058	**0.667**	Making bed	**0.796**	-0.043
Stiffness in the affected limb	0.015	**0.645**	Family activities	0.149	**0.418**
Hotness/increased temperature in the affected limb	0.082	**0.455**	Frustration	0.017	**0.72**
Redness in the affected limb	0.132	**0.389**	Sadness	-0.036	**0.771**
Numbness in the affected limb	-0.046	**0.438**	Guilt	-0.024	**0.714**
Burning in the affected limb	0.01	**0.36**	Concern	0.139	**0.687**
Stabbing in the affected limb	-0.119	**0.473**	Irritability	0.087	**0.743**
Tingling (pins and needles) in the affected limb	0.014	**0.505**	Fear	0.034	**0.708**
Fatigue in the affected limb	0.018	**0.579**	Anger	-0.013	**0.765**
Weakness in the affected limb	-0.034	**0.677**	Loneliness	0.022	**0.62**
Pocket of fluid developed	-0.065	**0.306**	Dependency	-0.024	**0.786**
Limited movement in shoulder	**0.756**	-0.005	Hopelessness	-0.139	**0.85**
Limited movement in elbow	**0.932**	-0.077	Anxiety	0.074	**0.764**
Limited movement in wrist	**0.982**	-0.033	Depression	-0.138	**0.898**
Limited movement in arm	**0.882**	-0.011	Self-perception	0.146	**0.441**
Limited movement in fingers	**0.833**	0.141	Sex life with partner	-0.126	**0.358**
			Emotional relationship with partner	-0.078	**0.511**
			Impact on work outside home	0.207	**0.335**

*Note.* O, Items from the occurrence subscale; D, Items from the distress subscale; bold numbers, items loaded above 0.30. BCLE-SEI = Breast Cancer–related Lymphedema Symptom Experience Index.

The three-factor solution for the distress subscale explained 54.9% of the total variance. The rotated solution did not exhibit a “simple structure.” Item 12, “Driving,” did not load adequately for any of the factors. The item was, therefore, dropped and the EFA was repeated. The PA analysis showed that dropping Item 12 led to a two-factor solution. However, the rotated solution for the two-factor solution did not exhibit a “simple structure.” Two of the items, “Tightness in the affected limb” and “How many times do you wake up at night because of your symptoms?” did not load onto either of the factors, and one item cross-loaded on both factors. Hence, these items were removed following further iterations of EFA to analyze different combinations of items, and it resulted in a simple two-factor structure: (a) physical-functional distress and (2) psychosocial distress. The impact subscale from the two-factor solution for the distress subscale explained 51% of the total variance (Table 2).

### Internal Consistency

The internal consistency of the BCLE-SEI Bengali symptom occurrence and symptom distress subscales was excellent (α = .92). The internal consistency of the dimensions ranged from high to excellent (α = .85 to .94, see Supplementary File 1).

### Reliability

The test–retest reliability (*n* = 10) of the overall BCLE-SEI Bengali was poor (ICC_2,1_ = .46). The test–retest reliability of the symptom distress was poor (ICC_2,1_ = 0.17), whereas the test–retest reliability of the symptom occurrence was moderate (ICC_2,1_ = .72, Table 3).

**Table 3.** The Test–Retest Reliability of BCLE-SEI-Bengali (n = 10).

Items	First Visit	Second Visit	ICC	95% CI (*p*)
	*M* ± *SD*	*M* ± *SD*	(Range)	
Total	50.2 ± 34.7	37.1 ± 20.5	.46	−0.2 to 0.8 (.76)
Symptom occurrence	18.3 ± 13.2	18.3 ± 13.2	.72	0.2 to 0.9 (.006)
Symptom distress	18.8 ± 11.7	18.8 ± 11.7	.17	−0.5 to 0.7 (.309)

*Note. SD* = standard deviation; ICC = intraclass correlation coefficient; CI = confidence interval.

### Hypothesis Testing

Spearman’s correlations between the dimensions of the BCLE-SEI-Bengali and scales of the EORTC-C30 and BR23 were examined (Table 4). In total, 92 correlations were checked based on the generic hypothesis, with 60 of them between the dimensions of BCLE-SEI-Bengali and scales of the EORTC-C30 and 32 of them between the dimensions of BCLE-SEI-Bengali and scales of the BR23. Nearly 72% of the correlations met the generic hypotheses proposed. Therefore, the construct validity of the BCLE-SEI-Bengali is met.

**Table 4.** Hypothesis Testing—Spearman’s Correlations Between the Dimensions of the BCLE-SEI-Bengali and the Scales of the EORTC-C30 and BR23 (n = 105–140).

EORTC-C30 and BR23	BCLE-SEI Bengali			
	TC^€^	MR^€^	PF^¥^	PsycF^¥^
QL2^£^	-0.28**	**-0.01**	-0.22*	-0.24**
PF2^£^	-0.32**	-0.24**	-.36**	-0.43**
RF2^£^	-0.35**	**-0.14**	-0.30**	-0.30**
EF^£^	-0.53**	**-0.14**	-0.32**	-0.70**
CF^£^	-0.45**	-0.17*	-0.38**	-0.45**
SF^£^	-0.41**	**-0.11**	-0.32**	-0.45**
FA^$^	0.42**	0.25**	0.39**	0.48**
NV^$^	0.18*	0.19*	0.19*	0.25**
**PA** ^$^	0.52**	0.35**	0.42**	0.34**
DY^$^	0.44**	0.37**	0.38**	0.37**
SL^$^	0.34**	0.20*	0.32**	0.42**
AP^$^	0.17*	*0.08*	0.23**	0.23**
CO^$^	0.29**	0.18*	*0.10*	0.28**
DI^$^	0.19*	*-0.10*	*0.06*	*0.11*
FI^$^	0.27**	*0.10*	0.25**	0.35**
BRBI^£^	-0.31**	*-0.04*	-0.23**	*-0.59* **
BRSEF^£^	-0.23**	*-0.05*	*0.05*	0.02
**BRSEE** ^£^	-0.19*	*0.03*	0.10	0.03
BRFU^£^	-0.31**	-0.09	-0.23**	-0.48**
**BRST** ^$^	0.36**	*0.16*	0.24**	0.51**
**BRBS** ^$^	0.52**	0.19*	0.33**	0.41**
BRAS^$^	0.58**	0.43**	0.29**	0.34**
**BRHL** ^$^	0.19*	-0.04	*0.03*	.31**

*Note.* BCLE-SEI = Breast Cancer–related Lymphedema Symptom Experience Index; QL2 = Global health status / QoL; PF2 = Physical functioning; RF2 = Role functioning; EF = Emotional functioning; CF = Cognitive functioning; SF = Social functioning; FA = Fatigue; NV = Nausea and vomiting; PA = Pain; DY = Dyspnea; SL = Insomnia; AP = Appetite loss; CO = Constipation; DI = Diarrhea; FI = Financial difficulties; BRBI = Body image; BRSEF = Sexual functioning; BRSEE = Sexual enjoyment; BRFU = Future perspective; BRST = Systemic therapy side effects; BRBS = Breast symptoms; BRAS = Arm symptoms; BRHL = Upset by hair loss; TC = Tissue changes; MR = Movement restriction; PF = Physical Function; PsycF = Psychosocial Function; £ = Functional scales; $ = Symptom scales; € = Dimensions of symptoms occurrence; ¥ = Dimensions of symptom distress; bold and/or italics = met hypothesis; bold and italic = similar and related constructs; bold = related but dissimilar constructs; plain and italics = not related and dissimilar constructs.

* Correlation is significant at the .05 level (two-tailed); **Correlation is significant at the .01 level (two-tailed).

### Known Group Validity (Discriminant Validity)

An independent-sample *t* test was conducted to determine if there were differences in symptom occurrence, symptom distress, and the total symptom experience (total score of BCLE-SEI-Bengali) between patients with and at risk of BCRL. Four different thresholds were used to determine whether BCRL was present, as there is a lack of consensus among the researchers about the thresholds. The symptom occurrence was higher in women with BCRL than at risk, irrespective of the different thresholds used (Supplementary file – Table 2). The mean differences of symptom occurrence subscale scores between women with or at risk of BCRL based on BIS ratio ≥ mean + 2*SD* and BIS ratio ≥ mean +3*SD*, and RAVD ≥ 5% and RAVD ≥ 10 % thresholds were 3.8 (95% CI = [0.2, 7.2], *p* = .036), 4.1 (95% CI = [0.3, 7.9], *p* = .035), 6.4 (95% CI = [3.2, 9.7], *p* = .0001) and 6.2 (95% CI = [2.3, 10.1], *p* = .002), respectively. The symptom distress score or the symptom experience index did not differ in women with or at risk of BCRL in any of the thresholds, except for the symptom experience index in women with BCRL based on RAVD-5% threshold 8.7 (95% CI = [1.1, 16.3], *p* = .025).

### BCRL Screening

The ROC curve analysis demonstrated sensitivity of using symptom count by BCLE-SEI-Bengali based on four commonly used BCRL thresholds: (a) BIS ratio ≥ mean + 2*SD*, (b) BIS ratio ≥ mean + 3*SD*, (c) RAVD ≥ 5%, and (d) RAVD ≥ 10% (Table 5). The volume-based thresholds (RAVD ≥ 5% and RAVD ≥ 10%) demonstrated higher AUC, Youden index and associated criterion than the BIS-based thresholds (BIS ratio ≥ mean + 2*SD* and ratio ≥ mean + 3*SD*). Symptom count (>7) by BCLE-SEI-Bengali to detect the four commonly used BCRL thresholds was excellent (<0.9), symptom count (>7) was found to be fair (AUC = 0.7, 95% CI = [0.6, 0.7]) with acceptable sensitivity and specificity (73.5 and 57.9) using the RAVD ≥10% as the reference threshold to detect BCRL.

**Table 5.** The Receiver Operating Characteristics Curve Analysis of Symptom Count Against Four Commonly Reported Thresholds for BCRL Detection.

Thresholds	AUC (95% CI)	Youden index J	Criterion (Symptom count)	Sensitivity (95% CI)	Specificity (95% CI)
BIS ≥ *M* + 2*SD*	0.60 (0.5 to 0.7)	0.2	>4	77.1 (62.7 to 88.0)	45.4 (36.2 to 54.8)
BIS ≥ *M* + 3*SD*	0.60 (0.5 to 0.7)	0.2	>4	78.9 (62.7 to 90.4)	44.2 (35.5 to 53.2)
RAVD ≥ 5%	0.70 (0.6 to 0.7)	0.3	>8	62.5 (48.5 to 75.1)	68.5 (59 to 77)
RAVD ≥ 10%	0.70 (0.6 to 0.7)	0.3	>7	73.5 (55.6 to 87.1)	57.9 (49 to 66.4)

*Note.* BCRL = breast cancer–related lymphedema; AUC = area under the curve; CI = confidence interval; BIS = bioimpedance spectroscopy ratio; RAVD = relative arm volume difference between affected and unaffected arms.

## Discussion

This study aimed to translate and validate the BCLE-SEI in the Bengali language and evaluate its psychometric properties. The results of this study showed that the BCLE-SEI-Bengali is a valid patient-reported measure to assess the symptom occurrence and distress in Bengali-speaking women with BCRL. The BCLE-SEI-Bengali was found to have good content validity, structural validity, internal consistency, and construct validity. The symptom count >7 symptoms using BCLE-SEI in Bengali was able to detect lymphedema. Similar to English, Chinese, and Spanish versions, the BCLE-SEI-Bengali is easy to use, and patients can use it as a home-based monitoring tool to assess their lymphedema and symptom status. In addition, using the BCLE-SEI-Bengali is cost-effective and time-efficient in resource-limited settings where high-tech devices, such as bioimpedance and perometry, are not available in routine clinical practice; such features make the BCLE-SEI-Bengali a choice for clinical use.

The BCLE-SEI-Bengali has met the required measurement properties. According to the COSMIN guidelines, sufficient content validity and internal structure (factor structure and internal consistency) are the minimum measurement properties for a PROM to be recommended (Prinsen et al., 2018). A large proportion of the participants agreed that the items and the responses in the BCLE-SEI-Bengali questionnaire were relevant to their symptom experience, easy to understand and comprehensive. Translation and validation of the BCLE-SEI in Chinese and Spanish also showed similar results (Cachero-Rodriguez et al., 2022; Shi et al., 2016).

Two conceptually meaningful factors were extracted for the occurrence of symptoms and the distress subscales of the BCLE-SEI-Bengali questionnaire. This factor structure was different from the proposed factor structure of the BCLE-SEI-English and the factor structures of the BCLE-SEI-Chinese and the BCLE-SEI-Spanish questionnaires (Cachero-Rodriguez et al., 2022; Fu et al., 2015; Shi et al., 2016). The symptom occurrence subscale of the Bengali version produced two distinct factors: (a) tissue changes and (b) movement restriction, whereas the Spanish BCEL-SEI produced one factor, similar to the proposed structure of the BCLE-SEI-English questionnaire. The authors of the BCLE-SEI-Spanish questionnaire explained that this was because all the items in the symptom occurrence were related to lymph fluid retention (Cachero-Rodriguez et al., 2022). However, the items in the factor movement restriction, such as restriction in the shoulder, elbow and finger movements, in the BCLE-SEI-Bengali could be due to lymph fluid retention or due to breast cancer treatment, including postoperative scarring and radiation-induced fibrosis (Ebaugh et al., 2011; Hidding et al., 2014).

The factor structure for the symptom distress subscale of the BCLE-SEI-Bengali was similar to that of the BCLE-SEI-Spanish questionnaire (Cachero-Rodriguez et al., 2022), whereas it differed from the Chinese and English versions (Fu et al., 2015; Shi et al., 2016). This may be due to the cultural differences in these populations. For example, an item in this subscale, “Driving,” did not load adequately to any of the factors because it is a common belief in Indian culture that driving is not for women. Dropping this item led to a two-factor solution. In addition, the item “How many times do you wake up at night because of your symptoms?” in the original English version was proposed to be an independent domain, and a single item was not amenable to factor extraction (Watkins, 2018). Hence, dropping these items led to a simple factor structure with a two-factor solution that was conceptually meaningful in the BCLE-SEI Bengali questionnaire.

Although COSMIN recommends confirmatory factor analysis to establish the factor structure of a PROM, we used EFA for two reasons: (a) factor structures of previously published BCLE-SEI questionnaires did not result in identical structures, and (b) the BCLE-SEI questionnaire has been translated into Bengali for the first time. It is advisable to confirm the factor structure in another set of Bengali women with or at risk of BCRL (Cachero-Rodriguez et al., 2022). However, the internal consistency of identified dimensions of the symptom occurrence and symptom distress subscales in this study ranged from high to excellent, similar to BCLE-SEI Chinese and Spanish (Cachero-Rodriguez et al., 2022; Shi et al., 2016). The BCLE-SEI-Bengali, therefore, could be used to capture the symptom experience in Bengali-speaking patients.

The construct validity of the BCLE-SEI Bengali was acceptable. The dimensions of the BCLE-SEI Bengali correlated well with the scales of the EORTC-QLQ-C30 and BR23 Bengali versions. As expected, many symptom occurrence and distress dimensions negatively correlated with the functional scales and positively correlated with the symptom scales of the EORTC-QLQ-C30 and BR23 Bengali questionnaires. This finding was similar to the BCLE-SEI Chinese (Shi et al., 2016), although the Chinese version was compared with SF-36, a generic QOL questionnaire.

## Limitations and Strengths of the Study

This study had limitations. First, a small sample of women (*n* = 10) were willing to participate in the test–retest procedure. Women were less willing to attend the repeat measure as this study was conducted at a Tertiary Cancer Hospital in Mumbai, which is far from West Bengal State, where Bengali is predominantly used. Hence, it was not practical for them to revisit the hospital again in such a short time only to participate in the reliability aspect of this study. This small sample may be the reason for the inconclusive result of the test–retest reliability (ICC = .46). In addition, the limited number of women (*n* = 47) with BCRL led to inconclusive findings on the known-group validity and ROC curve analysis (AUC ranged from 0.6 to 0.7) of the BCLE-SEI Bengali.

The strengths of the study included the use of the established protocols for the translation, back translation, and consolidation processes (Eremenco et al., 2018; International Test Commission, 2017). The validation study was guided by the COSMIN recommendations for the evaluation of psychometric properties. The use of objective measures for limb volume and lymph fluid level to validate BCRL symptoms is another strength.

## Conclusion

The results of this study demonstrated the reliability and validity of the BCLE-SEI-Bengali questionnaire, which could be used to assess the occurrence and distress of the symptoms in Bengali-speaking patients. The BCLE-SEI-Bengali questionnaire was found to have good content validity, structural validity, internal consistency, and construct validity. The use of symptom count by the BCLE-SEI-Bengali can be of great value in resource-limited settings. Studies with a larger sample size and a higher number of women with BCRL are recommended to establish the test–retest reliability, known group validity, and thresholds for discriminating between populations.

## Supplemental Material

sj-docx-1-tcn-10.1177_10436596251345338 – Supplemental material for Cross-Cultural Adaptation of the Breast Cancer and Lymphedema Symptom Experience Index in Bengali Supplemental material, sj-docx-1-tcn-10.1177_10436596251345338 for Cross-Cultural Adaptation of the Breast Cancer and Lymphedema Symptom Experience Index in Bengali by Vincent Singh Paramanandam, Elizabeth Dylke, Mei R. Fu, Anuradha Daptardar, Manali Kamat, Sarika Mahajan and Sharon Kilbreath in Journal of Transcultural Nursing

## References

[bibr1-10436596251345338] Aaronson N. K. Ahmedzai S. Bergman B. Bullinger M. Cull A. Duez N. J. Filiberti A. Flechtner H. Fleishman S. B. Dehaes J. Kaasa S. Klee M. Osoba D. Razavi D. Rofe P. B. Schraub S. Sneeuw K. Sullivan M. Takeda F. (1993). The European Organization for Research and Treatment of Cancer QLQ-C30: A quality-of-life instrument for use in international clinical trials in oncology. Journal of the National Cancer Institute, 85(5), 365–376. 10.1093/jnci/85.5.365 8433390

[bibr2-10436596251345338] Anand A. Mishra A. Damde H. Saxena A. Yadav S. K. Sharma D. (2022). Molecular profile and clinico-pathological characteristics of breast cancer in Central India: First Investigative Report. Indian Journal of Surgical Oncology, 13(2), 421–425. 10.1007/s13193-022-01502-0 35782821 PMC9240130

[bibr3-10436596251345338] Anthoine E. Moret L. Regnault A. Sébille V. Hardouin J.-B. (2014). Sample size used to validate a scale: A review of publications on newly-developed patient reported outcomes measures. Health and Quality of Life Outcomes, 12, 2. 10.1186/s12955-014-0176-2 PMC4275948 25492701

[bibr4-10436596251345338] Bhargavan R. V. Prasannan N. Krishna K. M. J. Augustine P. Cherian K. (2024). The role of level III dissection in locally advanced breast cancer following neoadjuvant chemotherapy-A prospective study. South Asian Journal of Cancer, 13(3), 170–176. 10.1055/s-0043-1777727 39410990 PMC11473130

[bibr5-10436596251345338] Bray F. Laversanne M. Sung H. Ferlay J. Siegel R. L. Soerjomataram I. Jemal A. (2024). Global cancer statistics 2022: GLOBOCAN estimates of incidence and mortality worldwide for 36 cancers in 185 countries. CA: A Cancer Journal for Clinicians, 74(3), 229–263. 10.3322/caac.21834 38572751

[bibr6-10436596251345338] Brorson H. Hoijer P. (2012). Standardised measurements used to order compression garments can be used to calculate arm volumes to evaluate lymphoedema treatment. Journal of Plastic Surgery and Hand Surgery, 46(6), 410–415. 10.3109/2000656X.2012.714785 23157502

[bibr7-10436596251345338] Bundred N. Foden P. Todd C. Morris J. Watterson D. Purushotham A. Bramley M. Riches K. Hodgkiss T. Evans A. Skene A. Keeley V ., & The Investigators of BEA/PLACE studies. (2020). Increases in arm volume predict lymphoedema and quality of life deficits after axillary surgery: A prospective cohort study. British Journal of Cancer, 123(1), 17–25. 10.1038/s41416-020-0844-4 32362658 PMC7341763

[bibr8-10436596251345338] Cachero-Rodriguez J. Menendez-Aller A. Fu M. R. Llaneza-Folgueras A. Fernandez-Alvarez M. M. Martin-Payo R. (2022). Psychometric properties of the Spanish version of Breast Cancer and Lymphedema Symptom Experience Index. Psicothema, 34(2), 291–298. 10.7334/psicothema2021.388 35485543

[bibr9-10436596251345338] Cornish B. H. Jacobs A. Thomas B. J. Ward L. C. (1999). Optimizing electrode sites for segmental bioimpedance measurements. Physiological Measurement, 20(3), 241–250. 10.1088/0967-3334/20/3/302 10475578

[bibr10-10436596251345338] DiSipio T. Rye S. Newman B. Hayes S. (2013). Incidence of unilateral arm lymphoedema after breast cancer: A systematic review and meta-analysis. The Lancet. Oncology, 14(6), 500–515. 10.1016/S1470-2045(13)70076-7 23540561

[bibr11-10436596251345338] Ebaugh D. Spinelli B. Schmitz K. H. (2011). Shoulder impairments and their association with symptomatic rotator cuff disease in breast cancer survivors. Medical Hypotheses, 77(4), 481–487. 10.1016/j.mehy.2011.06.015 21764521

[bibr12-10436596251345338] Eremenco S. Pease S. Mann S. Berry P. (2018). Patient-Reported Outcome (PRO) consortium translation process: Consensus development of updated best practices. Journal of Patient-Reported Outcomes, 2(1), Article 12. 10.1186/s41687-018-0037-6 PMC5934912 29757299

[bibr13-10436596251345338] Fancellu A. Giuliani G. Mulas S. Contini A. M. Ariu M. L. Sanna V. (2025). De-escalation of axillary treatment in early breast cancer-A narrative review of current trials [Review]. Translational Breast Cancer Research: A Journal Focusing on Translational Research in Breast Cancer, 6, 5. 10.21037/tbcr-24-45 39980812 PMC11836741

[bibr14-10436596251345338] Fu M. R. Axelrod D. Cleland C. M. Qiu Z. Guth A. A. Kleinman R. Scagliola J. Haber J. (2015). Symptom report in detecting breast cancer-related lymphedema. Breast Cancer: Targets and Therapy, 7, 345–352. http://dx.doi.org/10.2147/BCTT.S87854 26527899 10.2147/BCTT.S87854 PMC4621182

[bibr15-10436596251345338] Fu M. R. Axelrod D. Guth A. A. Rampertaap K. El-Shammaa N. Hiotis K. Scagliola J. Yu G. Wang Y. (2016). mHealth self-care interventions: Managing symptoms following breast cancer treatment. mhealth, 2, 28–28. 10.21037/mhealth.2016.07.03 27493951 PMC4970761

[bibr16-10436596251345338] Fu M. R. Liu B. W. Qiu J. M. Sun Y. L. Axelrod D. Guth A. Korth S. Kremer H. L. Wang Y. (2024). The effects of daily-living risks on breast cancer-related lymphedema. Annals of Surgical Oncology, 31(12), 8076–8085. 10.1245/s10434-024-15946-x 39090498 PMC11466982

[bibr17-10436596251345338] Fu M. R. McTernan M. L. Qiu J. M. Ko E. Yazicioglu S. Axelrod D. Guth A. Fan Z. Sang A. Miaskowski C. Wang Y. (2021). The effects of kinect-enhanced lymphatic exercise intervention on lymphatic pain, swelling, and lymph fluid level. Integrative Cancer Therapies, 20, 15347354211026757. 10.1177/15347354211026757 PMC8226364 34160294

[bibr18-10436596251345338] Fu M. R. Wang Y. Li C. Qiu Z. Axelrod D. Guth A. A. Scagliola J. Conley Y. Aouizerat B. E. Qiu J. M. Yu G. Van Cleave J. H. Haber J. Cheung Y. K. (2018). Machine learning for detection of lymphedema among breast cancer survivors. Mhealth, 4, 17. 10.21037/mhealth.2018.04.02 29963562 PMC5994440

[bibr19-10436596251345338] Gursen C. Dylke E. S. Moloney N. Meeus M. De Vrieze T. Devoogdt N. De Groef A. (2021). Self-reported signs and symptoms of secondary upper limb lymphoedema related to breast cancer treatment: Systematic review [Review]. European Journal of Cancer Care, 30(5), Article e13440. 10.1111/ecc.13440 33733550

[bibr20-10436596251345338] Hayes S. C. Dunn M. Plinsinga M. L. Reul-Hirche H. Ren Y. M. Laakso E. L. Troester M. A. (2022). Do patient-reported upper-body symptoms predict breast cancer-related lymphoedema: Results from a population-based, longitudinal breast cancer cohort study. Cancers, 14(23), Article5998. 10.3390/cancers14235998 PMC9740941 36497482

[bibr21-10436596251345338] Hidding J. T. Beurskens C. H. van der Wees P. J. van Laarhoven H. W. Nijhuis-van der Sanden M. W. (2014). Treatment related impairments in arm and shoulder in patients with breast cancer: A systematic review. PLOS ONE, 9(5), Article e96748. 10.1371/journal.pone.0096748 PMC4016041 24816774

[bibr22-10436596251345338] Ho P. J. Gernaat S. A. M. Hartman M. Verkooijen H. M. (2018). Health-related quality of life in Asian patients with breast cancer: A systematic review. BMJ Open, 8(4), Article e020512. 10.1136/bmjopen-2017-020512 PMC5914715 29678980

[bibr23-10436596251345338] Huang Y. Y. Toh P. Y. Hunt C. Lin J. T. W. Kamyab R. Ponniah A. K. (2022). Breast cancer treatment-related arm lymphoedema and morbidity: A 6-year experience in an Australian tertiary breast centre. Asia-Pacific Journal of Clinical Oncology, 18(1), 109–117. 10.1111/ajco.13523 33629541

[bibr24-10436596251345338] International Test Commission. (2017). The ITC guidelines for translating and adapting tests (2nd ed.). www.InTestCom.org

[bibr25-10436596251345338] Li K. Fu M. R. Zhao Q. Chen L. (2016). Translation and evaluation of Chinese version of the symptom experience index. International Journal of Nursing Practice, 22(6), 556–564. 10.1111/ijn.12464 27560042

[bibr26-10436596251345338] Lin Y. Xu Y. Wang C. J. Song Y. Huang X. Zhang X. H. Cao X. Sun Q. (2021). Loco-regional therapy and the risk of breast cancer-related lymphedema: A systematic review and meta-analysis. Breast Cancer, 28(6), 1261–1272. 10.1007/s12282-021-01263-8 34106427

[bibr27-10436596251345338] Lorenzo-Seva U. Ferrando P. J. (2006). FACTOR: A computer program to fit the exploratory factor analysis model. Behavior Research Methods, 38(1), 88–91. 10.3758/bf03192753 16817517

[bibr28-10436596251345338] Lorenzo-Seva U. Van Ginkel J. R. (2016). Multiple imputation of missing values in exploratory factor analysis of multidimensional scales: Estimating latent trait scores. Anales de Psicología, 32(2), 596–608. 10.6018/analesps.32.2.215161

[bibr29-10436596251345338] Luzaic K. Lachanas K. Vamvakopoulos K.-O. Sidiropoulos A. Vamvakopoulou D. Nomikos I. (2025). Axilla management in breast cancer surgery: Brief review and current practice recommendations [Review; Early access]. American Surgeon, 91(5):834–842. 10.1177/00031348251313529 39819186

[bibr30-10436596251345338] Macdonald E. R. Amorim N. M. L. Hagstrom A. D. Markovic K. Simar D. Ward R. E. Clifford B. K. (2024). Evaluating the effect of upper-body morbidity on quality of life following primary breast cancer treatment: A systematic review and meta-analysis [Review]. Journal of Cancer Survivorship, 18(5), 1517–1547. 10.1007/s11764-023-01395-0 37199900 PMC11424680

[bibr31-10436596251345338] Maratia S. Cedillo S. Rejas J. (2016). Assessing health-related quality of life in patients with breast cancer: A systematic and standardized comparison of available instruments using the EMPRO tool. Quality of Life Research, 25(10), 2467–2480. 10.1007/s11136-016-1284-8 27048496

[bibr32-10436596251345338] Nguyen J. Popovic M. Chow E. Cella D. Beaumont J. L. Chu D. DiGiovanni J. Lam H. Pulenzas N. Bottomley A. (2015). EORTC QLQ-BR23 and FACT-B for the assessment of quality of life in patients with breast cancer: A literature review. Journal of Comparative Effectiveness Research, 4(2), 157–166. 10.2217/cer.14.76 25825844

[bibr33-10436596251345338] Oliveira I. S. Menezes Costa L. d C. Cabral Fagundes F. R. Nunes Cabral C. M. (2015). Evaluation of cross-cultural adaptation and measurement properties of breast cancer-specific quality-of-life questionnaires: A systematic review [Review]. Quality of Life Research, 24(5), 1179–1195. 10.1007/s11136-014-0840-3 25391488

[bibr34-10436596251345338] Paramanandam V. S. Lee M. J. Kilbreath S. L. Dylke E. S. (2021). Self-reported questionnaires for lymphoedema: A systematic review of measurement properties using COSMIN framework. Acta Oncologica, 60(3), 379–391. 10.1080/0284186x.2020.1862422 33475033

[bibr35-10436596251345338] Parmar V. Badwe R. A. Hawaldar R. Rayabhattanavar S. Varghese A. Sharma R. Mittra I. (2005). Validation of EORTC quality-of-life questionnaire in Indian women with operable breast cancer. The National Medical Journal of India, 18(4), 172–177. 16252544

[bibr36-10436596251345338] Prinsen C. A. C. Mokkink L. B. Bouter L. M. Alonso J. Patrick D. L. de Vet H. C. W. Terwee C. B. (2018). COSMIN guideline for systematic reviews of patient-reported outcome measures. Quality of Life Research, 27(5), 1147–1157. 10.1007/s11136-018-1798-3 29435801 PMC5891568

[bibr37-10436596251345338] Qiu J. M. Fu M. R. Finlayson C. S. Tilley C. P. Payo R. M. Korth S. Kremer H. L. Lippincott C. L. R. (2024). Lymphatic pain in breast cancer survivors: An overview of the current evidence and recommendations. Women Child Nursing, 2(2), 33–38. 10.1016/j.wcn.2024.04.001 PMC11486487 39421196

[bibr38-10436596251345338] Ridner S. H. Dietrich M. S. (2015). Development and validation of the Lymphedema symptom and intensity survey-arm. Supportive Care in Cancer, 23(10), 3103–3112. 10.1007/s00520-015-2684-y 25752884 PMC4554806

[bibr39-10436596251345338] Sackey H. Johansson H. Sandelin K. Liljegren G. MacLean G. Frisell J. Brandberg Y. (2015). Self-perceived, but not objective lymphoedema is associated with decreased long-term health-related quality of life after breast cancer surgery. European Journal of Surgical Oncology, 41(4), 577–584. 10.1016/j.ejso.2014.12.006 25659877

[bibr40-10436596251345338] Shi S. Lu Q. Fu M. R. Ouyang Q. Liu C. Lv J. Wang Y. (2016). Psychometric properties of the Breast Cancer and Lymphedema Symptom Experience Index: The Chinese version. European Journal of Oncology Nursing, 20, 10–16. 10.1016/j.ejon.2015.05.002 26071198

[bibr41-10436596251345338] Sprangers M. A. G. Groenvold M. Arraras J. I. Franklin J. teVelde A. Muller M. Franzini L. Wiliams A. deHaes H. Hopwood P. Cull A. Aaronson N. K. (1996). The European Organization for Research and Treatment of Cancer breast cancer-specific quality-of-life questionnaire module: First results from a three-country field study. Journal of Clinical Oncology, 14(10), 2756–2768. 10.1200/jco.1996.14.10.2756 8874337

[bibr42-10436596251345338] Svensson B. J. Dylke E. S. Ward L. C. Black D. A. Kilbreath S. L. (2020). Screening for breast cancer-related lymphoedema: Self-assessment of symptoms and signs. Supportive Care in Cancer, 28(7), 3073–3080. 10.1007/s00520-019-05083-7 31641870

[bibr43-10436596251345338] Timmerman M. E. Lorenzo-Seva U. (2011). Dimensionality assessment of ordered polytomous items with parallel analysis. Psychological Methods, 16(2), 209–220. 10.1037/a0023353 21500916

[bibr44-10436596251345338] Togawa K. Ma H. Y. Smith A. W. Neuhouser M. L. George S. M. Baumgartner K. B. McTiernan A. Baumgartner R. Ballard R. M. Bernstein L. (2021). Self-reported symptoms of arm lymphedema and health-related quality of life among female breast cancer survivors. Scientific Reports, 11(1), Article 10701. 10.1038/s41598-021-89055-0 PMC8139966 34021179

[bibr45-10436596251345338] Wadasadawala T. Joshi S. Rath S. Popat P. Sahay A. Gulia S. Bhargava P. Krishnamurthy R. Hoysal D. Shah J. Engineer M. Bajpai J. Kothari B. Pathak R. Jaiswal D. Desai S. Shet T. Patil A. Pai T. . . . Badwe R. (2024). Tata Memorial Centre evidence based management of breast cancer. Indian Journal of Cancer, 61(Suppl. 1), S52–S79. 10.4103/ijc.ijc_55_24 38424682

[bibr46-10436596251345338] Watkins M. W. (2018). Exploratory factor analysis: A guide to best practice. Journal of Black Psychology, 44(3), 219–246. 10.1177/0095798418771807

[bibr47-10436596251345338] Wu R. Huang X. Dong X. Zhang H. Zhuang L. (2019). Obese patients have higher risk of breast cancer-related lymphedema than overweight patients after breast cancer: A meta-analysis. Annals of Translational Medicine, 7(8), 172. 10.21037/atm.2019.03.44 31168453 PMC6526274

