# Supplementary material for: Cross-Cultural Adaptation of the Breast Cancer and Lymphedema Symptom Experience Index in Bengali
Source: J Transcult Nurs. 2025 Jun 28;36(5):506–17. doi: 10.1177/10436596251345338 (PMC12335620; doi:10.1177/10436596251345338)
Supplement: sj-docx-1-tcn-10.1177_10436596251345338 – Supplemental material for Cross-Cultural Adaptation of the Breast Cancer and Lymphedema Symptom Experience Index in Bengali [file sj-docx-1-tcn-10.1177_10436596251345338.docx]

**Content validity questions**

**To be asked by the therapists**

Was each question of the BCLE-SEI understandable?

Was the scoring system clear?

Were all symptoms and distress related to your breast cancer/lymphedema mentioned in the BCLE-SEI?

Do you think that all items are relevant to you?

Do you want any other item must be added to this questionnaire?

Do you want any item must be deleted or modified from this questionnaire?

| **Supplementary Table 1**: The internal consistency of BCLE-SEI-Bengali (n = 168) | |
| --- | --- |
| **Items** | **Cronbach's Alpha** |
| Total | 0.94 |
| Symptom occurrence | 0.92 |
| Tissue changes | 0.89 |
| Movement restriction | 0.85 |
| Symptom distress | 0.92 |
| Physical/Functional Distress | 0.93 |
| Emotional/Social Distress | 0.92 |

| **Supplementary Table 2.** Known group validity | | | | | |
| --- | --- | --- | --- | --- | --- |
| **BIS** | **BCLE-SEI** | **LE status**  **(n)** | **Mean**  **(SD)** | **Mean_Diff**  **(95% CI, p-value)** | **Cohen's d**  **(95% CI)** |
| ≥ M+2SD | SymOcc | LE (48) | 14.2 (10.6) | **3.8** | **0.4** |
|  |  | At-risk (119) | 10.5 (10.4) | **(0.2 to 7.2, 0.036)** | **(0.02 to 0.7)** |
|  | SymDis | LE (48) | 20.6 (15) | -1.04 | **0.06** |
|  |  | At-risk (119) | 21.6 (16.8) | (-6.5 to 4.5, 0.709) | (-0.4 to 0.23) |
|  | Total | LE (48) | 34.8 (22.9) | 2.7 | 0.1 |
|  |  | At-risk (119) | 32.1 (24.1) | (-5.3 to 10.8, 0.504) | (0.1 to 0.7) |
| ≥ M+3SD | SymOcc | LE (38) | 14.7 (10.9) | **4.1** | **0.4** |
|  |  | At-risk (129) | 10.6 (10.3) | **(0.3 to 7.9, 0.035)** | **(0.03 to 0.8)** |
|  | SymDis | LE (38) | 21.5 (14.7) | 0.3 | 0.02 |
|  |  | At-risk (129) | 21.2 (16.7) | (-5.7 to 6.2, 0.924) | (-0.3 to 0.4) |
|  | Total | LE (38) | 36.2 (22.5) | 4.4 | 0.2 |
|  |  | At-risk (129) | 31.9 (24.1) | (-4.3 to 13, 0.319) | (-0.2 to 0.5) |
| **Volume** |  |  |  |  |  |
| RAVD ≥ 5% | SymOcc | LE (56) | 15.8 (11) | **6.4** | 0.6 |
|  |  | At-risk (111) | 9.1 (9.7) | **(3.2 to 9.7, 0.0001)** | (0.3 to 1) |
|  | SymDis | LE (56) | 22.8 (16.3) | 2.3 | 0.1 |
|  |  | At-risk (111) | 20.5 (16.3) | (-3 to 7.5, 0.394) | (-0.2 to 0.5) |
|  | Total | LE (56) | 38.6 (24) | **8.7** | **0.4** |
|  |  | At-risk (111) | 29.9 (23.2) | **(1.1 to 16.3, 0.025)** | **(0.04 to 0.7)** |
| RAVD ≥ 10% | SymOcc | LE (34) | 16.5 (11.1) | **6.2** | **0.6** |
|  |  | At-risk (133) | 10.3 (10.1) | **(2.3 to 10.1, 0.002)** | **(0.2 to 0.9)** |
|  | SymDis | LE (34) | 21.5 (16.2) | 0.3 | 0.01 |
|  |  | At-risk (133) | 21.2 (16.3) | (-5.9 to 6.5, 0.929) | (-0.4 to 0.4) |
|  | Total | LE (34) | 38 (24.4) | 6.5 | 0.3 |
|  |  | At-risk (133) | 31.5 (23.5) | (-2.5 to 15.5, 0.157) | (-0.10.70) |
| n, sample size; SD, standard deviation; CI, confidence interval; BIS, bioimpedance spectroscopy; SymOcc, symptom occurrence; SymDis, symptom distress; M, Mean; RAVD, relative arm volume difference; LE, Lymphedema. | | | | | |
